# P18 Antibiotic duration exceeds time to clinical stability in hospital acquired pneumonia

**DOI:** 10.1093/jacamr/dlaf230.025

**Published:** 2025-12-04

**Authors:** Elliott Lewis-Douglas, Jon Spedding, Sandhya Prem, Jamie Lupton, Daniel Weiand, Iain McCullagh, Tom Hellyer

**Affiliations:** Critical Care Department, Royal Victoria Infirmary, The Newcastle Hospitals NHS Foundation Trust, Newcastle, UK; Critical Care Department, Royal Victoria Infirmary, The Newcastle Hospitals NHS Foundation Trust, Newcastle, UK; Anaesthetics and Peri-operative Medicine, Royal Victoria Infirmary, The Newcastle Hospitals NHS Foundation Trust, Newcastle, UK; Integrated Laboratory Medicine Department, Freeman Hospital, The Newcastle Hospitals NHS Foundation Trust, Newcastle, UK; Department of Microbiology and Virology, Freeman Hospital, The Newcastle Hospitals NHS Foundation Trust, Newcastle, UK; Critical Care Department, Freeman Hospital, The Newcastle Hospitals NHS Foundation Trust, Newcastle, UK; Critical Care Department, Royal Victoria Infirmary, The Newcastle Hospitals NHS Foundation Trust, Newcastle, UK; Translational and Clinical Research Institute, Faculty of Medical Sciences, Newcastle University, Newcastle, UK

## Abstract

**Background:**

Hospital-acquired pneumonia (HAP) is a common indication for broad-spectrum antibiotics.^1^ Shorter course durations are becoming more widely accepted due to increasing evidence of non-inferior clinical effectiveness and safety compared to longer course lengths.^2,3^ Guidance for duration in HAP is often extrapolated from CAP and VAP evidence, and HAP lacks validated scoring systems to guide decision making.^4,5^ Time to clinical stability (TCS) criteria (heart rate <100, systolic blood pressure >90 mmHg, temperature <37.8°C, respiratory rate <25 and oxygen saturations >90%) have been shown to be a safe metric to guide duration decision making in CAP^2^ and effective in indicating risk of complications early in disease.^3^

**Objectives:**

To determine the TCS in relation to duration of antibiotics to identify whether there could be scope to further shorten antibiotic course length if guided by TCS criteria.

**Methods:**

We automated data-collection, screening the trust wide electronic patient record, during two non-consecutive weeks. Free-text antibiotic indication data were analysed to identify inpatients with chest-infection related terminology and prescribed antibiotics for more than 48 h. Manual searches excluded non-HAP respiratory infections. Further data collection included patient demographics; time to clinical stability; chest X-ray appearance; antibiotic prescriptions; Charlston comorbidity index; radiograph findings; and pathogens.

**Results:**

A total of 188 patients were identified in automated screening. After exclusions, 68 patients were included. The median (IQR) age was 76.5 years (62.3–82.5); 36.8% were female; and the median (IQR) Charlson Comorbidity Index was 6 (4–7.8). 34.4% of patients were surgical (23). The median (IQR) antibiotic duration was 5 (5–7) days whilst median time to clinical stability was 2 (0–3) days. For 92% of patients, clinical stability was maintained for >24 h. Twenty-five percent of patients had a change from initial antibiotic, which included equally escalations for deteriorations and de-escalations to oral antibiotics. Local prescribing guidelines were followed in 73% of patients. Piperacillin/tazobactam or a carbapenem was prescribed in 31% and 3% of patients, respectively as the initial choice of antibiotic. 68.8% of patients had chest radiograph findings recorded as positive. While an organism was identified in only 14% of patients, in 3% of patients *Pseudomonas aeruginosa*. was isolated. These two patients reached clinical stability at day 12 and 0.

**Conclusions:**

In this cohort receiving predominantly broad-spectrum antibiotics, TCS were reached much earlier than the prescribed antibiotic duration. We included patients based on recorded indication and treatment with antibiotics. The study is limited by diagnostic uncertainty as organisms were rarely identified and not all patients had radiological evidence of HAP. However, the frequent use of antibiotics without supporting diagnostic evidence, highlights the need for improved antibiotic stewardship in patients with suspected HAP. This study suggests that if antibiotics were tailored to achieving TCS, rather than the commonly used fixed duration approach, antibiotic course lengths could potentially be shortened.
